# 4295 The Impact of Social Determinants of Health on Hepatocellular Carcinoma Outcomes

**DOI:** 10.1017/cts.2020.281

**Published:** 2020-07-29

**Authors:** Lauren Devore Nephew, Susan Rawl, Archita Desai, Eric Orman, Marwan Ghabril, Kavish Patidar, Naga Chalasani

**Affiliations:** 1 Indiana Univeristy School of Medicine

## Abstract

OBJECTIVES/GOALS: Achieving therapy for hepatocellular carcinoma (HCC) involves navigating through a complex cascade of care. Non-HCC cancer mortality has been associated with social determinants of health outside of cancer specific risk. Our objective is to explore the impact of social determinants on HCC outcomes. METHODS/STUDY POPULATION: Patients with HCC were enrolled from 3 hospitals form June, 1 2019 to December 1, 2019. A chart review was done to collect information on liver disease severity and cancer stage. Patients were interviewed to collect information on the following: 1) socioeconomic status (income, education, insurance status, and employment status), 2) literacy (Rapid Estimate of Adult Literacy in Medicine (REALM-R) and Brief Health Literacy Screening Tool (BREIF)), 3) social support (Patent Reported Outcome Measurement Information System (PROMIS) instrumental and information support tool), 4) quality of life (PROMIS global and mental health tool), 5) substance abuse, and 6) linkage to care. RESULTS/ANTICIPATED RESULTS: Data compiled on the social determinants of health revealed (n = 35): 1) 60.0% of patients had incomes below $30,000 per year, 60.0% of patients had not gone past high school for education, and 8.6% had full time employment, 2) the average BREIF score was 10.3 (range 3-15)(4-12 indicate limited literacy). The average REALM-R score was 5.5 (range 0-8) (<6 indicate at risk for poor literacy), 3) patients had strong instrumental (T score 61.4±7.1) and information social support (T score 64.6±4.7) (mean T scores calibrated to a general population mean of 50), 4) patients had poor mental (T score 43.7 ±6.5) and physical quality of life (T score 46.6 ±9.9), 5) 25.7% of patients reported alcohol use in the past 90 days 6) 80.0% of patients reported that their doctor had spoken to them about liver transplantation. DISCUSSION/SIGNIFICANCE OF IMPACT: This patient population was well linked to care with good social support. However their literacy, socioeconomic status, mental and global health was poor and substance use history complex. Continued follow up of this cohort is planned to determine how these factors might impact their ability to navigate through the care cascade as well as survival.

